# Chipping in on clonal hematopoiesis

**DOI:** 10.18632/oncotarget.21472

**Published:** 2017-10-04

**Authors:** Nancy K. Gillis, Eric Padron

**Affiliations:** Eric Padron: Department of Malignant Hematology, H. Lee Moffitt Cancer Center and Research Institute, Tampa, FL, USA

**Keywords:** clonal hematopoiesis, CHIP, risk factor, myeloid neoplasms

Myeloid neoplasms are clonal diseases of hematopoietic stem or progenitor cells that result from molecular alterations that perturb cellular self-renewal, proliferation, and differentiation. As classified by the World Health Organization, myeloid neoplasms include myeloproliferative neoplasms (MPN), myelodysplastic syndromes (MDS), MPN/MDS overlap, and acute myeloid leukemia (AML), which can occur *de novo*, secondary to MDS/MPN, or after treatment with chemotherapy or radiation (therapy-related, T-MN). Epidemiological features of myeloid neoplasms include higher incidence in men, Caucasians, and increased frequency with age. Aside from these demographic criteria, there are currently no clear biomarkers or risk factors for predisposition to myeloid malignancies. The recent discovery of age-related clonal hematopoiesis [1, 2], commonly termed clonal hematopoiesis of indeterminate potential (CHIP), may narrow this critical knowledge gap.

CHIP is an idiopathic genetic event in which individuals harbor somatic mutations, primarily in genes associated with myeloid neoplasms (e.g., *DNMT3A, TET2,* and *ASXL1*), without overt signs of hematologic malignancy. Similar to myeloid neoplasms, CHIP mutations are most frequent in older individuals, men, and Caucasians [1-3]. The presence of CHIP is associated with poor outcomes, including a significantly increased risk of hematologic malignancies (HR 12.9), all-cause mortality (HR 1.4), and cardiovascular disease (HR 2.0) [1, 2]. We, and others, have demonstrated that individuals with CHIP mutations at the time of primary cancer are also at a significantly increased risk for T-MNs [4, 5]. Taken together, these findings suggest that CHIP mutations may represent an important biomarker for the development of myeloid neoplasms.

While we understand the basic demographics of individuals who have CHIP mutations, a complete understanding of who acquires CHIP has yet to be elucidated. One study reported a modest association (OR 1.37) with germline mutations in *TERT* (telomerase reverse transcriptase) [6]*.* Environmental factors, such as smoking and radiation, have also been associated with CHIP [1, 3]. However, it is also possible that CHIP occurs through stochastic errors in DNA replication. It has been hypothesized that cancer incidence rates correlate with stem cell turnover within the tissue of origin, especially for cancers without strong hereditary or environmental components [7]. In fact, a strong correlation (r^2^ = 0.80) was observed between total stem cell divisions during an average lifetime and lifetime risk for cancer of the corresponding tissue type [7]. Notably, cancers without known environmental risk factors, such as MDS and AML, had the strongest correlation with stem cell turnover. Perhaps this model of association between seemingly spontaneous cancers and stem cell division rate can help explain at least a portion of acquired CHIP mutations. If this hypothesis proves true for CHIP, efforts should be refocused toward secondary prevention (i.e., monitoring for early detection of progression to disease and early intervention), as primary prevention (i.e., avoidance of risk factors) would not impact stochastically acquired mutations.

A subsequent critical knowledge gap relates to what drives progression from CHIP to overt hematologic malignancy. Is CHIP a precursor for *de novo* MDS/AML in all cases? While the incidence of myeloid malignancies is significantly higher in individuals with CHIP, non-cancer patients with CHIP have only an approximately five percent absolute risk of developing a hematologic malignancy [2]. Contributors to progression could include hereditary, environmental, gene-specific factors (i.e., which “CHIP genes” are mutated), or stochastic events similar to that responsible for CHIP acquisition. Therefore, do the factors those drive CHIP development also drive progression? The observation that cancer patients of all ages have a significantly higher prevalence of CHIP than non-cancer cohorts [3, 4] provides some support for such a relationship.

In addition to its role in hematologic cancer development, CHIP may also have broader implications in health of the general population. Most notably, individuals with CHIP mutations have approximately two times the risk of cardiovascular disease when compared to individuals without CHIP mutations [2]. An elegant study demonstrated that this increased risk of cardiovascular disease is mediated through an inflammatory mechanism that could be inhibited, suggesting a future therapeutic intervention [8].

In summary, CHIP is an idiopathic event that is known to be associated with increased risk of hematologic, primarily myeloid, malignancies. The risk factors for development of myeloid neoplasms, CHIP, and progression of CHIP to overt disease, are currently unknown. Future studies exploring the possibility of stochastic, environmental (e.g., inflammation, drug exposures, etc.), and hereditary effects on development and progression of CHIP are warranted to inform implementation of this biomarker into clinical practice.

**Figure 1:**
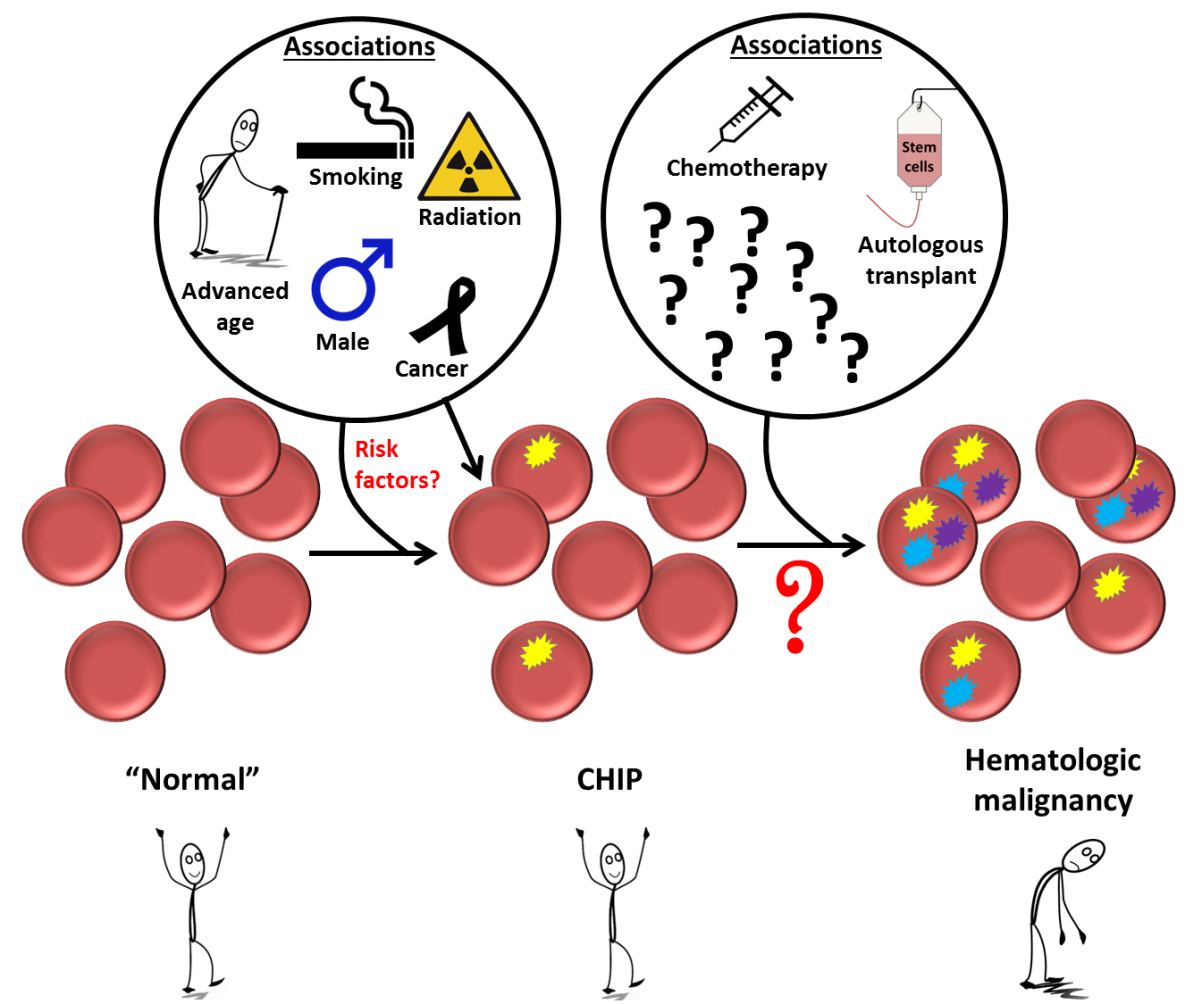
Factors that have been associated with clonal hematopoiesis of indeterminate potential (CHIP) and its progression to overt hematologic malignancy.
